# Cross‐Cultural Adaptation and Validation of KidSIM Attitude Towards Teamwork in Training Undergoing Designed Educational Simulation (ATTITUDES) in Undergraduate Healthcare Professionals

**DOI:** 10.1002/nop2.70499

**Published:** 2026-03-25

**Authors:** Simone Garzon, Elisa Campetti, Maria Panzeri, Simona Fumagalli, Antonella Nespoli, Anna Locatelli, Robert Fruscio, Marzia Serafini, Laura Antolini, Sabrina Valletta, Anna Adami, Mariachiara Bosco, Massimo Piergiuseppe Franchi, Stefano Uccella

**Affiliations:** ^1^ Unit of Obstetrics and Gynecology, Department of Surgery, Dentistry, Pediatrics, and Gynecology AOUI Verona Italy; ^2^ School of Medicine and Surgery University of Verona Verona Italy; ^3^ Unit of Obstetrics and Gynecology, Department of Mother and Child ASL Pescara Pescara Italy; ^4^ School of Medicine and Surgery University of Milano‐Bicocca Monza Italy; ^5^ Department of Obstetrics Fondazione IRCCS San Gerardo Dei Tintori Monza Italy

**Keywords:** attitudes, curriculum, interprofessional education, medical residents, midwifery students, simulation‐based training

## Abstract

**Aim:**

To translate and culturally adapt the ATTITUDES tool to the Italian context, assessing its validity and reliability in evaluating Italian students' attitudes towards interprofessional education (IPE) through simulations.

**Design:**

This is a cultural adaptation and psychometric pre‐post validation study.

**Methods:**

**T**he tool translation was conducted using the forward‐backward translation method. The subjects participated in interprofessional simulation sessions on obstetric emergencies in the delivery room, part of the standard training programme. Participants were invited to complete the Italian version of the ATTITUDES (I‐ATTITUDES) before and after the training.

**Results:**

A total of 81 midwifery students and medical residents participated in interprofessional simulation sessions. The average total score was significantly higher in the post‐simulation questionnaires compared to the pre‐simulation ones (137.84 vs. 143.03), indicating improved students' attitudes and perceptions about IPE using simulations as a learning modality. The Confirmatory Factor Analysis (CFA) suggested a five sub‐scale structure of attitudes toward teamwork in interprofessional training that fit the original ATTITUDES. The model showed good fit indices with Cronbach's alphas between 0.76 and 0.80 for four subscales and Cronbach's alpha = 0.46 for one subscale.

**Conclusions:**

The study confirms that the Italian version of the I‐ATTITUDES tool is valid.

**Implications for the Profession:**

The I‐ATTITUDES enables educators to assess and improve interprofessional simulation training by identifying specific learning needs, supporting tailored and effective teamwork education in clinical settings.

**Impact:**

This study addressed the lack of a validated Italian‐language tool to assess students' attitudes toward IPE through simulation. The study successfully translated and validated the I‐ATTITUDES questionnaire, demonstrating strong psychometric properties. It also found that the tool is sensitive to differences in students' educational backgrounds and prior clinical experiences. The I‐ATTITUDES tool can impact IPE in Italy, particularly among students and residents in obstetrics and midwifery programmes. Its adaptability suggests broader relevance for other healthcare disciplines.

**Reporting Method:**

STROBE.

## 1 Introduction

Interprofessional education (IPE) is becoming increasingly relevant in undergraduate programs (Colonnello et al. 2023; Oudbier et al. 2024). IPE is defined by the World Health Organization (WHO) as an experience in which students from two or more professions learn with, from and about each other, aiming to develop knowledge, skills and attitudes that promote interprofessional team competencies (Global Forum on Innovation in Health Professional Education, Board on Global Health, Institute of Medicine 2013).

Despite the growing interest in IPE, few tools have been translated and validated to measure attitudes toward simulation‐based IPE in obstetrics and gynaecology and other health professional contexts.

Simulations‐based IPE has been shown to enhance learning by providing students from different professions with structured, realistic experiences in a safe environment. Students engage in simulation‐based concrete experiences (Kolb 1984), designed to replicate real practice contexts, to learn with, from and about each other (UK Centre for the Advancement of Interprofessional Education 1997).

Simulation has been demonstrated to improve knowledge, team cohesion, leadership, problem‐solving, communication skills and overall patient care (Morese and Ragusa 2015; Labrague et al. 2018). Moreover, the inclusion of midwives, who now play a more senior role in hospital settings, is increasingly recognised as essential in interprofessional training initiatives.

## 2 Background

Clinical contexts today rely on the ability of different health professionals to work seamlessly together, especially in fields such as obstetrics and gynaecology, where there is always a risk of emergencies involving the mother and/or newborn (Avery et al. 2020). Providing care for women and their families during childbirth necessitates strong teamwork between midwives and medical professionals. As midwifery becomes increasingly academic, it is both essential and practical for midwifery and medical students to begin collaborating early in their education. Interprofessional education (IPE), defined by CAIPE as a process in which students ‘learn with, from and about each other,’ supports the development of such collaborative competencies (UK Centre for the Advancement of Interprofessional Education 1997).

IPE initiatives promoted by the American College of Nurse‐Midwives and the American College of Obstetricians and Gynecologists, has demonstrated multiple benefits (Avery et al. 2020), including improved understanding of interdisciplinary teamwork, enhanced technical skills and greater confidence in communication and leadership. Additionally, clinical simulation has proven particularly effective in promoting collaborative learning and improving the care of women during childbirth and the post‐partum period (Juschka et al. 2024).

In this scenario, understanding healthcare students' attitudes toward IPE should be a priority to identify unmet needs and align curricula to expected learning outcomes. To align curricula with expected learning outcomes, understanding healthcare students' attitudes toward IPE should be a priority. For this purpose, several validated instruments exist with good psychometric properties, such as the Readiness for Interprofessional Learning Scale (RIPLS) (Parsell and Bligh 1999; McFadyen et al. 2005), the Attitudes Toward Health Care Teams Scale (ATHCTS) (Heinemann et al. 1999), the Collaborative Healthcare Interdisciplinary Relationship (CHIRP) (Hollar et al. 2012), the Jefferson Scale of Attitudes toward Physician‐Nurse Collaboration (JSAPNC) (Hojat et al. 1999), and the Interprofessional Attitudes Scale (IPAS) (Norris et al. 2015). Additional tools validated in Italy, such as the Italian version of RIPLS (Spada et al. 2022) and the Italian validation of the Chiba Interprofessional Competency Scale (CICS29) (Tonarelli et al. 2020), further contribute to the assessment of interprofessional competencies. However, these instruments do not specifically assess attitudes toward simulation as a learning modality for IPE. The ‘KidSIM ATTITUDES’ instrument (Attitude Toward Teamwork In Training Undergoing Designed Educational Simulation), developed and validated in Canada in 2012 (Sigalet et al. 2012), uniquely evaluates healthcare students' perceptions of simulation‐based IPE, alongside standard international domains. Educational strategies that enhance both technical and non‐technical competencies.

## 3 Study Aim

Because the ‘ATTITUDES’ tool is not available in the Italian language, this study aimed to translate and culturally adapt the ‘ATTITUDES’ tool to the Italian context, assessing its validity and reliability in measuring Italian students' attitudes toward IPE by simulations. This will support the design of interprofessional training programs based on simulation tailored to the country's cultural and educational specificities.

## 4 Methods

### 4.1 Study Design

This was a cultural adaptation and psychometric pre‐post validation study conducted in two phases: (1) The Italian version of the ATTITUDES instrument was developed through a process of translation and verification of semantic, cultural and content equivalence, following established guidelines (Wild et al. 2005); (2) The validation of the instrument involved collecting and analysing data to determine the psychometric properties of the Italian version of ATTITUDES.

### 4.2 Description of the ATTITUDES Questionnaire

The ‘ATTITUDES’ questionnaire was developed to measure the perceptions and attitudes of students toward IPE and teamwork, with simulation as a learning modality (Sigalet et al. 2012). It consists of 30 items rated on a five‐point Likert scale ranging from 1 (‘strongly disagree’) to 5 (‘strongly agree’). The items are organised into five domains: Relevance of IPE (7 items); Relevance of simulation (5 items); Communication (8 items); Situation awareness (4 items); Roles and responsibilities (6 items). A higher total score on the questionnaire indicates greater readiness for collaborative learning with students from other professions. The instrument has shown excellent psychometric properties, with a Cronbach's alpha of 0.95 and a percentage of total explained variance of 61.6%. This reliability and validity of this tool make it suitable for evaluating the perceptions of healthcare students in diverse simulation‐based educational contexts.

### 4.3 Translation and Cross‐Cultural Adaptation of the ATTITUDES

The ATTITUDES instrument was translated and culturally adapted from English to Italian, following the ‘Principles of Good Practice for the Translation and Cultural Adaptation Process for Patient‐Reported Outcomes’ guidelines (Wild et al. 2005). Permission to use the ATTITUDES instrument was sought from the Canadian researcher, Elaine Sigalet, who was responsible for developing the tool.

Translation and cross‐cultural adaptation of ATTITUDES was conducted by two research team members with previous experience of psychometric scale cross‐cultural adaptation and validation (Nespoli et al. 2018, 2021, 2024; Fumagalli et al. 2022). The procedure used for translation was the ‘forward‐back translation.’ The original version was translated into two independent Italian versions by two healthcare workers who were experts in translation and living in the UK but were native Italian speakers. A third independent translator compared and reconciled the two translations, producing a final Italian version of the ATTITUDES tool. Next, a native English‐speaking healthcare professional with a good knowledge of the Italian language and who was unfamiliar with the ATTITUDES tool performed the back translation. The project manager and the author of the original tool, Elaine Sigalet, reviewed the back translation product to confirm the equivalence of the two forms. During translation, harmonisation was carried out during all stages of the process to ensure the validity of inter‐translation and enable a reliable pool of controlled data.

### 4.4 Setting and Participants

Eligible subjects included midwifery students and obstetrics and gynaecology residents from the University of Verona and the University of Milano‐Bicocca, who had simulation‐based IPEincorporated into their study programs. Midwifery students were enrolled in the third year of their 3‐year undergraduate program, while obstetrics and gynaecology residents were in the second to fifth year of their 6‐year specialty training program. These differences in training seniority were taken into account when interpreting the study findings.

Participants in small groups performed in‐person interprofessional simulation sessions focused on obstetric emergencies in the delivery room. The scenarios included shoulder dystocia, assisted breech delivery and operative vaginal delivery with the obstetric vacuum. Each training session involved 9–12 participants, divided into three groups of 3–4 individuals each. A station was set up for each scenario with three different settings and three distinct trainers/tutors. Participants spent an hour and a half at each station, beginning with a theoretical section followed by practical simulations and role‐playing exercises. Simulation materials included advanced mannequins, pelvic trainers and medical devices such as gloves, lubricating gel, ultrasound machines, Foley catheters and Kiwi Omnicups. Following the initial hour and a half, groups rotated to the next station, ensuring that by the end of the day, each group had experienced all three simulations.

The planning and organisation of the interprofessional simulation educational interventions were consistent across both universities.

Participants were invited to participate in the study via email, which included an information sheet, informed consent to participate and details regarding the processing of personal data, which were sent one week before the scheduled training event. In the 24 h preceding the simulation, participants completed an online questionnaire based on the Italian translation of the ‘ATTITUDES’ survey tool using an electronic platform (Data S1). The same questionnaire was administered again within 72 h post‐simulation. Data were linked only to email addresses; no personal identifiers such as names, matriculation numbers or telephone numbers were collected.

To assess the validity and reliability of the instrument, a minimum of 10 respondents per item was required for the largest subscale of the original instrument (Barton and Peat 2014). In the ATTITUDES instrument, the ‘Communication’ subscale contains eight items. Therefore, a sample size of 80 subjects was deemed adequate to achieve the study's objectives.

### 4.5 Ethical Consideration

All participants voluntarily took part and were fully informed about the study's purpose; informed consent was obtained before accessing the survey. To ensure the confidentiality of respondents, all communication was managed by a researcher who was unaffiliated with the Midwifery Bachelor's Degree Program and the Obstetrics and Gynecology Residency Program of the University of Verona and the University of Milano‐Bicocca. This researcher held no official position within these institutions. The pseudo‐anonymization key was accessible only to the external researcher.

### 4.6 Statistical Analysis

Standard descriptive statistics were used to summarise sample characteristics. The validity of the Italian version of the ATTITUDES was initially assessed through confirmatory factorial analysis (CFA) to verify the original structure identified by the authors (Sigalet et al. 2012) within the Italian context. The CFA was conducted using a Diagonally Weighted Least Squares (DWLS) estimator, recommended for instruments with ordinal items, such as the Likert scale. The structural model was evaluated with absolute and incremental fit measures. Absolute fit measures were the chi‐square, whose robustness decreases as the sample size and the number of items increase (ideal threshold value > 0.05), and the Root Mean Square Error of Approximation (RMSEA), which is considered the best informative fit index (optimal threshold value > 0.07). Incremental fit indices included the Comparative Fit Index (CFI), which takes care of small sample size (ideal threshold value > 0.90 and > 0.95 for small sample size), and the Tucker–Lewis Index (TLI), which is not affected by the sample size (ideal threshold value > 0.90) (Dash and Paul 2021). To improve structural validity, modification indices were analysed to estimate the amount of which the Chi‐square would be enhanced if a single parameter were removed from the model; moreover, modification indices were evaluated with the theoretical consideration of each item to ensure the improvement of the conceptual and statistical characteristics (a restrictive threshold value > 10 was identified to avoid bias due to the small sample size). Then, the internal reliability of the Italian ATTITUDES questionnaire was computed for each dimension performing Cronbach's alpha. To detect disagreement rates between pre‐ and post‐intervention scores, we calculated how many participants had pre‐intervention scores different from post‐intervention scores (pre‐intervention scores lower or higher than post‐intervention scores) in each item; moreover, we computed agreement rates between pre‐ and post‐intervention scores, detecting how many participants had equal pre‐ and post‐intervention scores. Paired *t*‐tests were performed to describe differences between pre‐intervention and post‐intervention for the total score and each subscale. Stratification for professional status (midwifery students and medical residents) and previous experience of emergencies, defined as the experience of at least an emergency, including operative vaginal delivery, shoulder dystocia or breech birth, was conducted to describe differences in perception and attitudes toward interprofessional simulation as learning modalities. All tests were two‐sided; a *p*‐value < 0.05 was considered statistically significant.

Statistical analyses were performed using Stata/MP 18.0 and R (version 4.3.2).

## 5 Results

### 5.1 Description of the Sample Characteristics

The study included 81 participants, of whom 64.2% (*n* = 52) were midwifery students and 35.8% (*n* = 29) were medical residents in obstetrics and gynaecology. A stratified analysis according to profession showed that among the 52 midwifery students, 88.46% had previously participated in mannequin‐based simulation, 13.46% had prior exposure to IPE activities, and 92.31% had already encountered an obstetric emergency in the delivery room. Among the 29 residents, 46.43% had previous experience with mannequin‐based simulation, 17.86% with IPE and 64.29% had encountered an emergency. These distributions are reported in Table 1.

**TABLE 1:** Distribution of participants' characteristics overall and stratified by profession (Midwifery students and Medical residents).

	Overall *n* = 81		Midwifery students *n* = 52 (64.20%)		Medical resident *n* = 29 (35.80%)	
	*n*	%	*n*	%	*n*	%
Previous experience with mannequin‐based simulation training (Yes)	59	72.84	46	88.46	13	46.43
Previous experience of IPE (Yes)	69	85.19	7	13.46	5	17.86
Previous experience of emergency (Yes)	66	81.48	48	92.31	18	64.29
IPE: Interprofessional education.						

### 5.2 Psychometric Characteristics of I‐ATTITUDES

**FIGURE 1:**
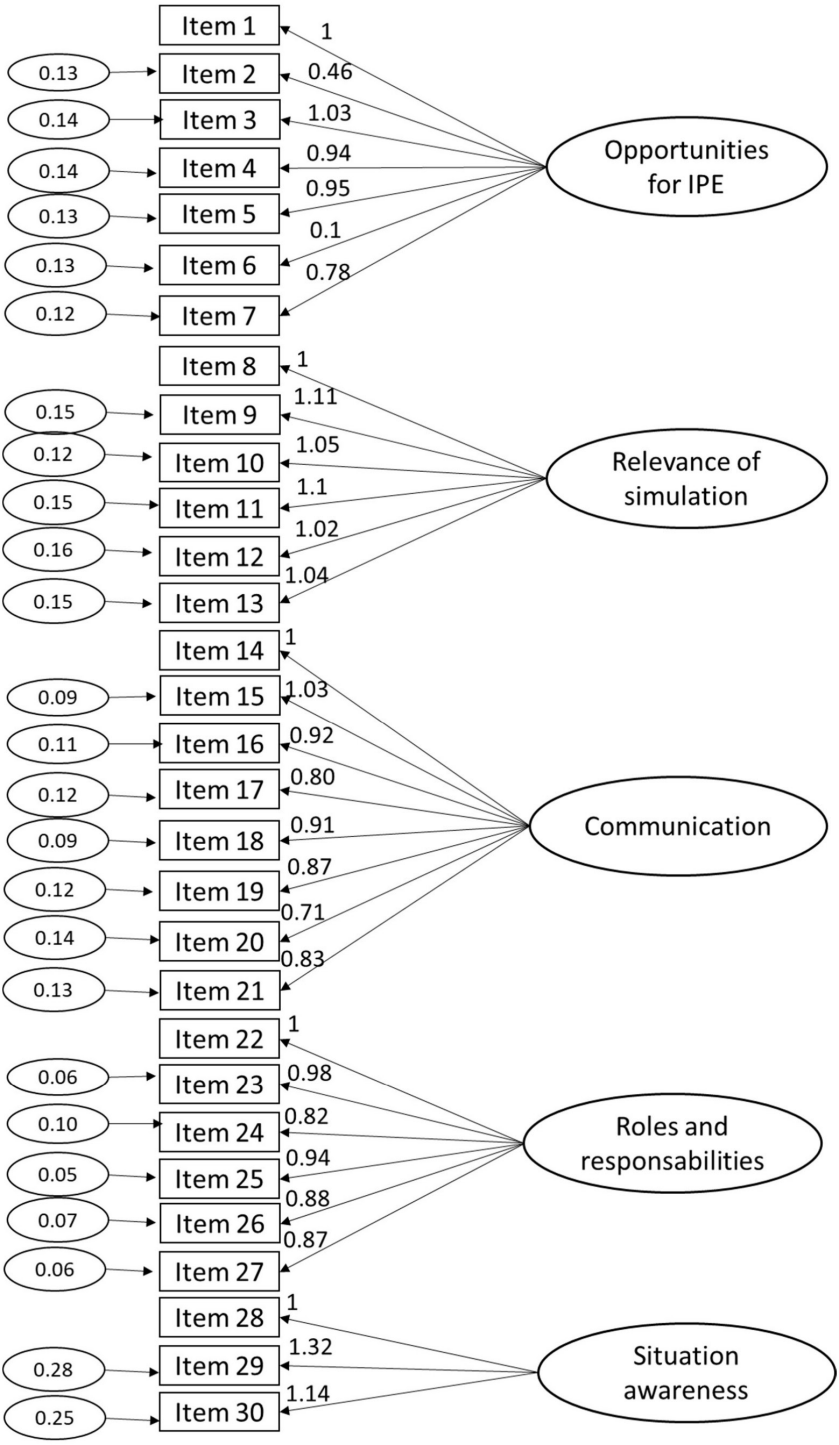
Factor structure of I‐ATTITUDES computed from confirmatory structural analysis.

The CFA of the original ATTITUDES questionnaire structure, using the DWLS estimator, assessed the structural validity of the Italian version of the instrument. Model parameter estimates (Figure 1) and fit indices indicated a good fit for the model: Chi‐square = 425.85 (*p*‐value 0.137), RMSEA = 0.031, CFI = 0.995, and TLI = 0.995. The modification indices supported the validity of the original five‐domain model not reaching the pre‐defined threshold (McFadyen et al. 2005). Reliability was demonstrated across four subscales of I‐ATTITUDES, with Cronbach's alpha values of 0.76 for ‘Opportunities for IPE,’ 0.76 for ‘Relevance of Simulation,’ 0.77 for ‘Communication’ and 0.80 for ‘Roles and Responsibilities. Reliability for the ‘Situation awareness' subscale was not established, with a Cronbach's alpha of 0.46.

### 5.3 Descriptive Statistics and Significance Testing of I‐ATTITUDES

The distribution of Likert scale values for each item and agreement and disagreement rates between pre‐ and post‐intervention questionnaires is fully described and represented in Table S1 and Figure S1. The total average score was significantly higher in the post‐simulation questionnaires compared to the pre‐simulation ones (pre‐intervention: 137.84 versus post‐intervention: 143.03, *p*‐value < 0.001); similar results were observed across all subscales (Table 2). For midwifery students, the total score for each domain was significantly higher in the post‐simulation questionnaire than in the pre‐simulation ones (Table 3). Conversely, among medical residents, significant differences were observed only in the total score (*p*‐value = 0.045) and the communication subscale (*p*‐value = 0.03) (Table 3).

**TABLE 2:** Distribution of total score; ‘Opportunities for IPE,’ ‘Relevance of simulation’, ‘Communication,’ ‘Roles and responsibilities’ and ‘Situation awareness’ subscales; 95% Confidence Interval (95% CI); and *p*‐value of pre‐intervention and post‐intervention questionnaires.

		Mean	95% CI	*p*
Total score	Pre	137.84	135.67; 140.00	0.000
	Post	143.03	141.07; 144.98	
Opportunities for IPE	Pre	32.57	32.01; 33.13	0.007
	Post	33.53	32.96; 34.10	
Relevance of simulation	Pre	27.74	27.23; 28.25	0.002
	Post	28.52	28.01; 29.03	
Communication	Pre	36.84	36.18; 37.49	0.000
	Post	38.23	37.68; 38.77	
Roles and responsibilities	Pre	27.26	26.69; 27.83	0.000
	Post	28.60	28.14; 29.07	
Situation awareness	Pre	13.47	13.17; 13.76	0.000
	Post	14.20	13.94; 14.45	

Abbreviation: IPE, interprofessional education.

**TABLE 3:** Distribution of total score; ‘Opportunities for IPE,’ ‘Relevance of simulation,’ ‘Communication,’ ‘Roles and responsibilities’ and ‘Situation awareness’ subscales; 95% Confidence Interval (95% CI) and *p*‐value of pre‐intervention and post‐intervention questionnaires within midwifery students and medical residents.

		Midwifery students (*n* = 52)			Medical residents (*n* = 29)		
		Mean	95% CI	*p*	Mean	95% CI	*p*
Total score	Pre	135.67	132.90; 138.45	< 0.001	141.86	138.92; 144.79	0.045
	Post	141.94	139.56; 144.32		145.04	141.70; 148.37	
Opportunities for IPE	Pre	32.17	31.41; 32.93	0.046	33.28	32.58; 33.98	0.051
	Post	33.17	32.37; 33.97		34.17	33.56; 34.78	
Relevance of simulation	Pre	27.25	26.61; 27.89	0.004	28.62	27.86; 29.38	0.266
	Post	28.25	27.57; 28.93		29.00	28.31; 29.69	
Communication	Pre	36.40	35.56; 37.24	< 0.001	37.64	36.65; 38.64	0.030
	Post	37.98	37.36; 38.60		38.68	37.65; 39.71	
Roles and responsibilities	Pre	26.60	25.87; 27.32	< 0.001	28.45	27.72; 29.18	0.319
	Post	28.40	27.91; 28.90		28.97	28.01; 29.92	
Situation awareness	Pre	13.25	12.87; 13.63	< 0.001	13.86	13.43; 14.29	0.167
	Post	14.13	13.88; 14.39		14.31	13.76; 14.86	

Abbreviation: IPE, Interprofessional Education.

For participants with prior emergency experience, attitudes and perceptions toward IPE with simulation as the learning modality were higher after the simulation in both the total score and each domain (Table 4). In contrast, participants without prior emergency experience showed no significant differences in average scores for the IPE subscale (*p*‐value = 0.109) and the roles and responsibilities subscale (*p*‐value = 0.087) between pre‐ and post‐simulation (Table 4).

**TABLE 4:** Distribution of total score; ‘Opportunities for IPE,’ ‘Relevance of simulation,’ ‘Communication,’ ‘Roles and responsibilities’ and ‘Situation awareness’ subscales; 95% Confidence Interval (95% CI); and *p*‐value of pre‐intervention and post‐intervention questionnaires within participants who had a previous experience of emergency and who had not.

		Previous experience of emergency (*n* = 66)			No previous experience of emergency (*n* = 15)		
		Mean	95% CI	*p*	Mean	95% CI	*p*
Total score	Pre	137.22	134.81; 139.62	< 0.001	140.21	135.06; 145.37	0.019
	Post	142.22	140.02; 144.41		146.29	142.10; 150.47	
Opportunities for IPE	Pre	32.59	31.96; 33.22	0.039	32.50	31.14; 33.86	0.109
	Post	33.39	32.74; 34.05		34.07	33.03; 35.11	
Relevance of Simulation	Pre	27.62	27.06; 28.19	0.017	28.14	26.91; 29.37	0.026
	Post	28.32	27.72; 28.91		29.36	28.63; 30.09	
Communication	Pre	36.69	35.97; 37.42	< 0.001	37.36	35.72; 39.00	0.045
	Post	38.06	37.46; 38.67		38.86	37.59; 40.12	
Roles and responsibilities	Pre	27.00	26.37; 27.63	< 0.001	28.29	27.10; 29.47	0.087
	Post	28.42	27.90; 28.95		29.36	28.36; 30.36	
Situation awareness	Pre	13.36	13.03; 13.69	< 0.001	13.93	13.26; 14.59	0.045
	Post	14.09	13.80; 14.38		14.64	14.20; 15.08	

Abbreviation: IPE, Interprofessional Education.

## 6 Discussion

The development of the Italian version of the ATTITUDES questionnaire (I‐ATTITUDES) was successful. The translation and cultural adaptation did not show discrepancies between the back‐translation and the original instrument. Psychometric evaluation of the I‐ATTITUDES reported validity and reliability consistent with the original version. Confirmatory factor analysis supported grouping the 30 items into the same original five subscales as established by Sigalet E. et al. (Sigalet et al. 2012). Only the internal consistency of the ‘Situational Awareness’ subscale was lower than acceptable limits. Although this subscale was also the lowest for the original version, this result may suggest further investigations on the validity of this subscale or a possible revision of associated questions for the Italian version.

Our study also assessed the sensitivity of the I‐ATTITUDES questionnaire by comparing pre‐ and post‐training responses. Consistent with the original validation study, our results showed a significant positive shift in the perceptions and attitudes of students toward IPE by simulation following simulation‐based training. Moreover, our findings suggest a sensitivity of the I‐ATTITUDES in identifying differences in the background of students and associated differences in the impact of simulation interventions. Baseline scores of midwifery students were lower than those of residents in gynaecology and obstetrics. Additionally, midwifery students exhibited a significantly greater score improvement from the pre‐test to the post‐test for all subscales. In contrast, residents in gynaecology and obstetrics showed significance only in the ‘Communication’ subscale and a borderline significance in the ‘Relevance of IPE’ subscale. The higher experience in teamwork of medical residents provided by everyday clinical practice may increase their baseline attitudes toward IPE and simulations, consequently creating less room for improvement. Midwifery students with limited clinical practice periods (approximately 4 months annually) may initially possess a lower baseline awareness of the value of simulations. This is consistent with previous literature supporting the notion that attitudes and perceptions toward IPE vary significantly based on the experience level of the student (Sigalet et al. 2012; Al‐Eisa et al. 2016; Katoue et al. 2022).

Furthermore, differences in prior exposure to mannequin‐based simulation, IPE activities and real obstetric emergencies between midwifery students and residents may also have contributed to the different levels of responsiveness observed across the I‐ATTITUDES subscales. Residents generally reported higher levels of previous clinical and simulation experience, which may have resulted in more stable baseline attitudes and reduced the extent of detectable change. Conversely, midwifery students, who had more limited prior exposure, may have been more receptive to the simulation‐based IPE intervention, demonstrating significant improvements across all domains.

In our study, the term *background* specifically refers to the students' level of education, year in training and prior exposure to clinical emergencies and IPE or simulation activities, which differed substantially between midwifery students and medical residents.

An additional different finding concerns the impact of prior experience with obstetric emergencies. Participants who had experienced at least one emergency in their clinical practice showed a more marked improvement than those who had never encountered such situations. This observation aligns closely with the principles of adult learning theory, particularly the concept that adults learn more effectively when they can connect new knowledge to prior practical experiences (Merriam and Bierema 2013; Knowles et al. 2020; Knowles 1984). According to Knowles' theory of andragogy, adult learners are motivated by relevance and immediate application of the knowledge they acquire. Students with prior exposure to stressful and urgent clinical situations are likely to recognise the applicability of simulation‐based training, which allows them to refine and reinforce their practical skills in a controlled environment. The experiential learning model proposed by Kolb further supports this interpretation, highlighting how concrete experience serves as the foundation for reflective learning and active experimentation (Kolb 1984). In our study, participants with previous experience in emergencies appeared to engage more actively with the simulation scenarios, using their past experiences as a basis for reflection and skill enhancement. These students likely benefited from the opportunity to practice decision‐making, teamwork and problem‐solving in scenarios that closely resembled real‐life situations they had already encountered. In contrast, participants without prior exposure to obstetric emergencies demonstrated less significant changes in their IPE‐related scores, particularly in understanding roles and responsibilities or situational awareness. This highlights the need for tailored educational strategies for students with limited clinical experience, such as pre‐simulation theoretical preparation and stepwise exposure to emergency scenarios. Gradual integration of theoretical and practical learning may help build the foundational knowledge and confidence required to maximise the benefits of simulation‐based training.

Designing simulations that consider both the background of students in teamwork and practical experiences directly connected with simulation‐based training can enhance the effectiveness of learning. By addressing these gaps, educators can create a more effective approach to interprofessional simulation‐based education. Furthermore, the I‐ATTITUDES tool may provide practical insights to guide the design and refinement of simulation‐based training programs. By identifying specific subscale strengths and weaknesses with a pre‐intervention assessment, such as ‘Roles and Responsibilities’ or ‘Situational Awareness,’ educators can develop targeted interventions to address these areas. For instance, simulations could emphasise interprofessional role clarity and collaboration if a cohort demonstrates weaker performance in understanding roles. With post‐intervention assessment, the I‐ATTITUDES tool can serve as a feedback mechanism, helping educators monitor progress and adjust curricula to align with evolving student needs. The flexibility of the I‐ATTITUDES tool indicates that it can be effectively applied in different interprofessional educational settings beyond the obstetric field. By identifying specific strengths and areas for improvement within interprofessional competencies, the instrument can guide the design and refinement of simulation‐based educational interventions and support the continuous development of interprofessional curricula.

## 7 Strengths and Limitations

A key strength of our study is the inclusion of obstetrics and gynaecology residents and midwifery students across two university centres with different training curricula. While the inclusion of two university centres adds some heterogeneity to the sample, the generalisability of our findings remains limited. Broader validation across different regions and health professional groups would be required to fully establish the applicability of the instrument at a national level. However, a limitation of the study is the adherence to the minimum sample size, which might restrict the robustness of the results, although it was estimated following standardised methods. The sub‐analysis for the obstetrics and gynaecology resident cohort, with absent statistical significance in some domains, may reflect a Type II error due to the small subpopulation size. This limitation highlights the need for further studies with larger cohorts of medical residents to confirm these findings. Finally, the validation of the I‐ATTITUDES questionnaire was specific to the context of IPE in obstetrics, which may limit its generalisability to other contexts. Expanding future research to additional healthcare disciplines could improve the applicability of the instrument across diverse interprofessional settings.

## 8 Conclusions

The present study translated and validated the original version of the ATTITUDES tool within the Italian context, focusing specifically on simulation‐based interprofessional education. The findings indicate that the I‐ATTITUDES questionnaire shows appropriate psychometric properties and appears to be a sensitive instrument for assessing students' perceptions, attitudes and behaviours before and after simulation‐based IPE interventions. These results may assist educators in designing, refining and strengthening simulation‐based interprofessional learning activities to better respond to students' evolving educational needs. In particular, lower scores in specific domains of the I‐ATTITUDES may help identify areas of the curriculum that require targeted simulation‐based interventions. However, the generalisability of these findings remains limited, as the tool has been tested only within obstetric contexts and in a relatively homogeneous sample. Further research involving larger and more diverse health professional populations and varied educational settings is needed to confirm these results and evaluate the instrument's applicability more broadly. Future studies may also explore the tool's usefulness in supporting long‐term interprofessional learning and its potential implications for clinical practice and patient outcomes.

## Author Contributions

**Simone Garzon:** conceptualization; project administration; resources; supervision; validation; writing – original draft preparation; writing – review and editing. **Elisa Campetti:** conceptualization; investigation; writing – original draft preparation; writing – review and editing. **Maria Panzeri:** data curation; formal analysis; visualisation; writing – original draft preparation; writing – review and editing. **Simona Fumagalli:** conceptualization; resources; writing – review and editing. **Antonella Nespoli:** writing – review and editing. **Anna Locatelli:** writing – review and editing. **Robert Fruscio:** writing – review and editing. **Marzia Serafini:** investigation; writing – review and editing. **Laura Antolini:** data curation; formal analysis; visualisation; writing – original draft preparation; writing – review and editing. **Sabrina Valletta:** investigation; writing – review and editing. **Anna Adami:** conceptualization; resources; writing – review and editing. **Mariachiara Bosco:** investigation; writing – review and editing. **Massimo Piergiuseppe Franchi:** writing – review and editing. **Stefano Uccella:** conceptualization; project administration; resources; supervision; validation; writing – review and editing.

## Funding

The authors have nothing to report.

## Ethics Statement

The study was approved by the Human Research Ethics Committee of Verona University in April 2022 (Protocol 24.R1/2021).

## Conflicts of Interest

The authors declare no conflicts of interest.

## Supporting information


**Figure S1:** Percentages’ distribution of disagreement between pre intervention and post intervention score.

**Table S1:** Distribution of Likert scale values for each item between pre and post‐intervention questionnaires.

**Data S1:** Supplemental Digital Content (SDC). I‐ATTITUDES.

## Data Availability

The data that support the findings of this study are available from the corresponding author upon reasonable request.
