# Supplementary material for: Cross‐Cultural Adaptation and Validation of KidSIM Attitude Towards Teamwork in Training Undergoing Designed Educational Simulation (ATTITUDES) in Undergraduate Healthcare Professionals
Source: Nurs Open. 2026 Mar 25;13(3):e70499. doi: 10.1002/nop2.70499 (PMC13098093; doi:10.1002/nop2.70499)

**Figure S1:**

Percentages’ distribution of disagreement between pre intervention and post intervention score within item, score pre intervention higher than post intervention and score pre intervention lower than post intervention, and percentages’ distribution of agreement within item, score pre intervention equal to score post intervention. Items are displayed on y axis.
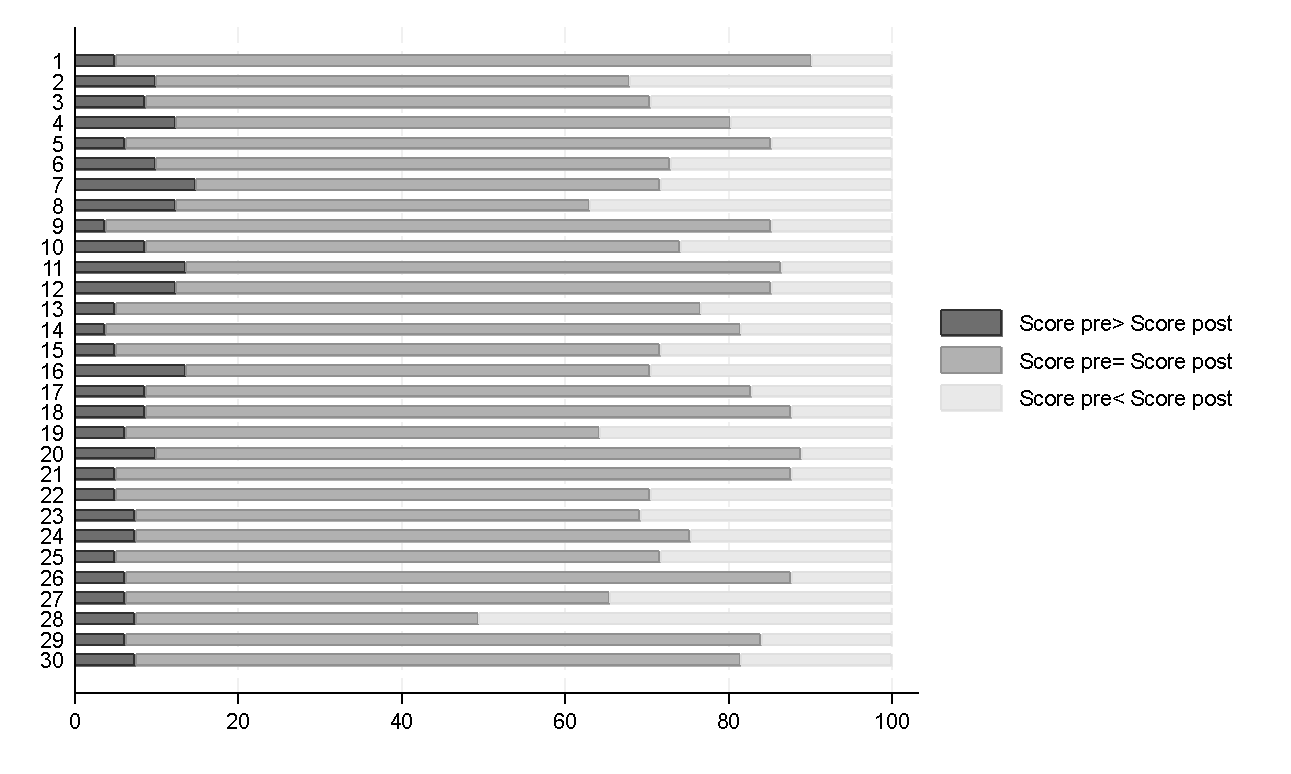

Supplement: Supplementary file 1 — Figure S1: Percentages’ distribution of disagreement between pre intervention and post intervention score. [file NOP2-13-e70499-s001.docx]
