# Supplementary material for: Cross‐Cultural Adaptation and Validation of KidSIM Attitude Towards Teamwork in Training Undergoing Designed Educational Simulation (ATTITUDES) in Undergraduate Healthcare Professionals
Source: Nurs Open. 2026 Mar 25;13(3):e70499. doi: 10.1002/nop2.70499 (PMC13098093; doi:10.1002/nop2.70499)
Supplement: Supplementary file 2 — Table S1: Distribution of Likert scale values for each item between pre and post‐intervention questionnaires. [file NOP2-13-e70499-s003.docx]

**Table S1:**

Distribution of Likert scale values for each item between pre and post-intervention questionnaires.

|  | score 1 | | score 2 | | score 3 | | score 4 | | score 5 | |
| --- | --- | --- | --- | --- | --- | --- | --- | --- | --- | --- |
|  | n | % | n | % | n | % | n | % | n | % |
| **Pre intervention** | | | | | | | | | | |
| 1 | 0 | 0.00 | 0 | 0.00 | 0 | 0.00 | 10 | 12.35 | 71 | 87.65 |
| 2 | 0 | 0.00 | 1 | 1.23 | 5 | 6.17 | 30 | 37.04 | 45 | 55.56 |
| 3 | 0 | 0.00 | 0 | 0.00 | 5 | 6.17 | 25 | 30.86 | 51 | 62.96 |
| 4 | 0 | 0.00 | 0 | 0.00 | 5 | 6.17 | 15 | 18.52 | 61 | 75.31 |
| 5 | 0 | 0.00 | 0 | 0.00 | 2 | 2.47 | 16 | 19.75 | 63 | 77.78 |
| 6 | 0 | 0.00 | 1 | 1.23 | 3 | 3.70 | 21 | 25.93 | 56 | 69.14 |
| 7 | 0 | 0.00 | 1 | 1.23 | 2 | 2.47 | 27 | 33.33 | 51 | 62.96 |
| 8 | 0 | 0.00 | 1 | 1.23 | 7 | 8.64 | 32 | 39.51 | 41 | 50.62 |
| 9 | 0 | 0.00 | 0 | 0.00 | 2 | 2.47 | 20 | 24.69 | 59 | 72.84 |
| 10 | 0 | 0.00 | 0 | 0.00 | 4 | 4.94 | 25 | 30.86 | 52 | 64.20 |
| 11 | 0 | 0.00 | 0 | 0.00 | 1 | 1.23 | 14 | 17.28 | 66 | 81.48 |
| 12 | 0 | 0.00 | 0 | 0.00 | 1 | 1.23 | 18 | 22.22 | 62 | 76.54 |
| 13 | 0 | 0.00 | 1 | 1.23 | 6 | 7.41 | 26 | 32.10 | 48 | 59.26 |
| 14 | 0 | 0.00 | 0 | 0.00 | 0 | 0.00 | 17 | 20.99 | 64 | 79.01 |
| 15 | 0 | 0.00 | 0 | 0.00 | 6 | 7.41 | 26 | 32.10 | 49 | 60.49 |
| 16 | 0 | 0.00 | 1 | 1.23 | 11 | 13.58 | 24 | 29.63 | 45 | 55.56 |
| 17 | 0 | 0.00 | 0 | 0.00 | 1 | 1.23 | 17 | 20.99 | 63 | 77.78 |
| 18 | 0 | 0.00 | 0 | 0.00 | 6 | 7.41 | 28 | 34.57 | 47 | 58.02 |
| 19 | 0 | 0.00 | 2 | 2.47 | 15 | 18.52 | 30 | 37.04 | 34 | 41.98 |
| 20 | 1 | 1.23 | 0 | 0.00 | 1 | 1.23 | 9 | 11.11 | 70 | 86.42 |
| 21 | 0 | 0.00 | 0 | 0.00 | 0 | 0.00 | 14 | 17.28 | 67 | 82.72 |
| 22 | 0 | 0.00 | 0 | 0.00 | 7 | 8.64 | 29 | 35.80 | 45 | 55.56 |
| 23 | 0 | 0.00 | 0 | 0.00 | 7 | 8.64 | 31 | 38.27 | 43 | 53.09 |
| 24 | 0 | 0.00 | 0 | 0.00 | 2 | 2.47 | 28 | 34.57 | 51 | 62.96 |
| 25 | 0 | 0.00 | 0 | 0.00 | 6 | 7.41 | 27 | 33.33 | 48 | 59.26 |
| 26 | 0 | 0.00 | 0 | 0.00 | 1 | 1.23 | 10 | 12.35 | 70 | 86.42 |
| 27 | 0 | 0.00 | 0 | 0.00 | 13 | 16.05 | 25 | 30.86 | 43 | 53.09 |
| 28 | 0 | 0.00 | 4 | 4.94 | 18 | 22.22 | 33 | 40.74 | 26 | 32.10 |
| 29 | 0 | 0.00 | 0 | 0.00 | 3 | 3.70 | 13 | 16.05 | 65 | 80.25 |
| 30 | 0 | 0.00 | 0 | 0.00 | 2 | 2.47 | 20 | 24.69 | 59 | 72.84 |
| **Post intervention** | | | | | | | | | | |
| 1 | 0 | 0 | 0 | 0.00 | 1 | 1.23 | 5 | 6.17 | 75 | 92.59 |
| 2 | 0 | 0 | 0 | 0.00 | 5 | 6.17 | 14 | 17.28 | 62 | 76.54 |
| 3 | 0 | 0 | 0 | 0.00 | 3 | 3.70 | 11 | 13.58 | 67 | 82.72 |
| 4 | 0 | 0 | 0 | 0.00 | 2 | 2.47 | 14 | 17.28 | 65 | 80.25 |
| 5 | 0 | 0 | 0 | 0.00 | 1 | 1.23 | 11 | 13.58 | 69 | 85.19 |
| 6 | 0 | 0 | 0 | 0.00 | 3 | 3.70 | 9 | 11.11 | 68 | 83.95 |
| 7 | 0 | 0 | 0 | 0.00 | 5 | 6.17 | 16 | 19.75 | 60 | 74.07 |
| 8 | 0 | 0 | 1 | 1.23 | 2 | 2.47 | 20 | 24.69 | 58 | 71.60 |
| 9 | 0 | 0 | 0 | 0.00 | 1 | 1.23 | 12 | 14.81 | 68 | 83.95 |
| 10 | 0 | 0 | 0 | 0.00 | 4 | 4.94 | 10 | 12.35 | 67 | 82.72 |
| 11 | 0 | 0 | 0 | 0.00 | 1 | 1.23 | 14 | 17.28 | 66 | 81.48 |
| 12 | 0 | 0 | 0 | 0.00 | 4 | 4.94 | 12 | 14.81 | 65 | 80.25 |
| 13 | 0 | 0 | 0 | 0.00 | 7 | 8.64 | 11 | 13.58 | 63 | 77.78 |
| 14 | 0 | 0 | 0 | 0.00 | 0 | 0.00 | 5 | 6.17 | 76 | 93.83 |
| 15 | 0 | 0 | 0 | 0.00 | 2 | 2.47 | 13 | 16.05 | 66 | 81.48 |
| 16 | 0 | 0 | 1 | 1.23 | 4 | 4.94 | 22 | 27.16 | 54 | 66.67 |
| 17 | 0 | 0 | 0 | 0.00 | 1 | 1.23 | 10 | 12.35 | 70 | 86.42 |
| 18 | 0 | 0 | 0 | 0.00 | 3 | 3.70 | 14 | 17.28 | 64 | 79.01 |
| 19 | 0 | 0 | 2 | 2.47 | 7 | 8.64 | 16 | 19.75 | 56 | 69.14 |
| 20 | 0 | 0 | 0 | 0.00 | 1 | 1.23 | 9 | 11.11 | 71 | 87.65 |
| 21 | 0 | 0 | 0 | 0.00 | 1 | 1.25 | 6 | 7.50 | 73 | 91.25 |
| 22 | 0 | 0 | 0 | 0.00 | 1 | 1.23 | 19 | 23.46 | 61 | 75.31 |
| 23 | 0 | 0 | 0 | 0.00 | 2 | 2.47 | 19 | 23.46 | 60 | 74.07 |
| 24 | 0 | 0 | 0 | 0.00 | 4 | 4.94 | 10 | 12.35 | 67 | 82.72 |
| 25 | 0 | 0 | 0 | 0.00 | 3 | 3.70 | 13 | 16.05 | 65 | 80.25 |
| 26 | 0 | 0 | 0 | 0.00 | 1 | 1.23 | 5 | 6.17 | 75 | 92.59 |
| 27 | 0 | 0 | 0 | 0.00 | 5 | 6.17 | 15 | 18.52 | 61 | 75.31 |
| 28 | 0 | 0 | 1 | 1.23 | 5 | 6.17 | 26 | 32.10 | 49 | 60.49 |
| 29 | 0 | 0 | 0 | 0.00 | 1 | 1.23 | 8 | 9.88 | 72 | 88.89 |
| 30 | 0 | 0 | 0 | 0.00 | 1 | 1.23 | 14 | 17.28 | 66 | 81.48 |
