# Supplementary material for: Cross‐Cultural Adaptation and Validation of KidSIM Attitude Towards Teamwork in Training Undergoing Designed Educational Simulation (ATTITUDES) in Undergraduate Healthcare Professionals
Source: Nurs Open. 2026 Mar 25;13(3):e70499. doi: 10.1002/nop2.70499 (PMC13098093; doi:10.1002/nop2.70499)
Supplement: Supplementary file 3 — Data S1: Supplemental Digital Content (SDC). I‐ATTITUDES. [file NOP2-13-e70499-s002.pdf]

Questionario I-ATTITUDES: Attitude Toward Teams in Training Undergoing Designed Educational Simulation	Scala Likert				
**Opportunità dell’educazione interprofessionale**	1	2	3	4	5
1. L'apprendimento con altri professionisti è importante per la collaborazione					
2. Le opportunità per apprendere con altri professionisti dovrebbero essere una priorità nella mia formazione					
3. Vorrei avere più opportunità di apprendimento con altri professionisti					
4. L'apprendimento condiviso con altri membri del team migliorerà la mia capacità di comprendere i problemi clinici					
5. Le opportunità di apprendimento interprofessionale miglioreranno gli esiti di salute delle persone assistite					
6. Le opportunità di fare simulazioni con altri professionisti possono cambiare l'attitudine al lavoro in team					
7. L’apprendimento con altri professionisti prima di essere abilitati all’esercizio della professione è importante per lo sviluppo delle future relazioni inter-professionali					
**Importanza delle simulazioni**					
8. La simulazione facilita le opportunità di cambiare le attitudini					
9. La simulazione è un buon metodo (contesto) per l’apprendimento con altri professionisti della salute					
10. La simulazione è un buono strumento per esercitare le capacità decisionali del team					
11. La pratica consapevole può migliorare le capacità di prendere decisioni cliniche					
12. Le opportunità di esercitarsi in team possono aiutare gli studenti ad apprendere i ruoli inter-professionali					
13. Le opportunità di apprendere con altri professionisti della salute hanno aumentato la mia conoscenza dei loro ruoli					
**Comunicazione**					
14. Tutti gli studenti dovrebbero imparare come si lavora nel contesto dei teams di assistenza sanitaria					
15. I team leader dovrebbero fornire frequenti aggiornamenti sulle persone assistite agli altri membri del team					
16. I team leader dovrebbero incoraggiare i membri del team a porre domande					
17. La comunicazione all'interno del team è importante quanto le abilità tecniche					
18. I membri del team che prestano immediata assistenza alle persone dovrebbero verbalizzare le loro attività ad alta voce					
19. I membri del team dovrebbero parafrasare o ripetere le istruzioni per chiarirne la comprensione					
20. La comunicazione all'interno del team è importante per la sicurezza della persona assistita					
21. I ruoli dei membri del team (non leader) sono importanti per il buon funzionamento del team tanto quanto una buona leadership					
**Ruoli e Responsabilità**					
22. Monitorare cosa ciascun membro del team sta facendo è importante per ottimizzare la sicurezza della persona assistita					

23. Il monitoraggio migliorerà la comprensione da parte degli altri membri del team del mio ruolo nell'assistenza sanitaria alle persone assistite					
24. Il lavoro in team mi fornirà un riscontro per migliorare la mia capacità di fornire assistenza ottimale					
25. Il lavoro in team mi aiuterà a riconoscere il modo migliore per aiutare gli altri membri del team a completare le loro attività					
26. È importante per i membri del team chiedere assistenza se necessitano di supporto per completare un'attività					
27. Il lavoro in team permette la flessibilità dei ruoli in situazioni critiche					
**Consapevolezza della situazione**					
28. Parlerò francamente se percepisco un problema indipendentemente da chi potrebbe essere coinvolto					
29. L'assistenza alla persona è migliorata quando tutti i membri del team hanno una comprensione condivisa della valutazione e del trattamento					
30. I team leader dovrebbero fornire frequenti aggiornamenti degli esiti di salute delle persone assistite per mantenere i membri del team orientati sui bisogni delle persone					
